# Quack Advertisements

**Published:** 1881-09

**Authors:** 


### Quack Advertisements.

The most despicable of all literary frauds and liars are those employes of nostrum mongers who assume the character of physicians and write down the profession for the purpose of writing up quackery. Of course this is done anonymously, for no respectable physician could make such a fool of himself. It is a part of the quack-medicine machinery. The inventor of a preparation to be put on the market as a new and valuable discovery, employs some smart scribbler who is otherwise a miserable hack, to puff it. For this purpose no fiction is too gross and mendacious. Assuming the character of a respectable physician of great experience, the hired penman throws himself soul and body into the service of his master and stops at no falsehood which his skill in that line may suggest. Nor has he any difficulty to get a hearing before the public. He knows that newspapers in general have their price, and that "religious" periodicals are not above the captivating influence of money. We have just observed in a weekly religious journal published in the East, a long article of the kind referred to, in which the writer represents himself as a "reporter" giving the experience of an old and well-known doctor, who was cured of some disease (not of lying) by a certain quack "kidney cure," when his professional brethren had failed to relieve him. Of course the "Doctor" was manufactured in the brain of the reporter, and yet the story will gain credence with a large per cent. of readers, particularly as it appears in a religious paper, and not in the usual form of an advertisement.

There is greatly needed a sense of personal responsibility on the part of journalists in regard to the advertisements which they publish. At present this is entirely wanting. There is no one to stand between the conductors of a journal and the public. Not only are editors and managers irresponsible in this respect, but their direct endorsement of the most palpable falsehoods is often purchasable with money. The agent of some unprincipled nostrum-monger or abortionist goes to the newspaper manager with a proposition substantially as follows: "I want you to publish for me this budget of lies—forged certificates of cure, disguised abortion notices, and so forth. I want it inserted, not as an advertisement, but under another head, so that people will think the editors have written or indorsed it. I want to buy, not only the use of your columns, but, for the time being, that of the names, character and souls of all the editors and others concerned with your paper. What is your charge?" The representative of the press answers: "Our people don't much like to father such a job as that. One of them is a preacher, you know, and they are nearly all strictly religious. We shall have to charge you just double price for putting that stuff in as you want it. Our men can't sell their consciences for nothing." And so the bargain is made.

We do not pretend to say that this is the literal method of transacting the business, nor that every journalist is guilty of it. But the picture is not much overdrawn, and the practice is injurious and disgraceful, and ought to be abandoned.—*Pacific Medical and Surgical Journal.*

